# Wnt signaling as a regulator of memory T cells: implications for CAR-T cell therapy

**DOI:** 10.3389/fimmu.2026.1843548

**Published:** 2026-05-13

**Authors:** Tatiana Fourfouris, Ki Jun Lee, Samantha Hurwitz, Asher Ahdoot, Alexander Lee, Maiah Zarrabi, Michael Kahn, Yong-Mi Kim

**Affiliations:** 1 Kim Lab, Children’s Hospital Los Angeles, Department of Pediatrics, Division of Hematology and Oncology, Keck School of Medicine, University of Southern California, Los Angeles, CA, United States; 2 Kahn Lab, City of Hope Beckman Research Institute, Cancer Biology and Molecular Medicine, Duarte, CA, United States

**Keywords:** CAR-T cell persistence, CAR-T cells, LEF1, T cells, TCF1 (TCF7), Wnt/β-catenin signaling, memory T cells

## Abstract

Chimeric antigen receptor T-cell (CAR-T) therapy has achieved impressive remission rates in hematologic cancers, but long-term efficacy remains limited by insufficient CAR-T cell persistence. T cell factor 1 (TCF1) and lymphoid enhancer binding factor 1 (LEF1), transcription factors well known for their role in downstream Wnt/β-catenin signaling, have been found to regulate transcriptional and epigenetic memory programming important for CAR-T cell persistence and favorable patient outcomes. Activation of the Wnt/β-catenin in endogenous T cells was found to arrest effector differentiation and promote the formation of cluster of differentiation (CD) 8+ memory stem cells, characterized by strong proliferative and recall potential, key traits of persisting memory cells. Genetically engineered CAR-T cells are subject to the same transcriptional and epigenetic factors that govern memory development in endogenous T cells, providing a strong rationale for applying scientific findings from basic T cell biology to CAR-T cell engineering. With this in mind, recent studies have shown that there is clinical potential for Wnt-directed approaches to improve CAR-T cell memory phenotypes, persistence, and exhaustion. Here we review the role of Wnt/β-catenin signaling in T cell development and memory formation, examine clinical evidence linking Wnt/TCF1 activity to CAR-T cell persistence and patient outcomes, and discuss emerging genetic, epigenetic, and pharmacological strategies used to target this pathway in CAR-T cell manufacturing.

## 1 Introduction

The Nusse and Varmus 1982 discovery of Wnt signaling molecules (a combination of “Wingless” a gene from Drosophila, and “*Int-1*,” a mouse proto-oncogene) has since sparked extensive research into the crucial role of this evolutionarily conserved signaling pathway in embryonic development, cell fate and migration, and tissue homeostasis (1–7). However, given its well-established role in oncogenesis, the Wnt/β-catenin pathway has also been an attractive target for therapeutic intervention across cancer models (8, 9). Today, cancer treatment strategies encompass a variety of immunotherapeutic approaches, including chimeric antigen receptor T-cell (CAR-T) therapy. First approved in 2017 for the treatment of pediatric and young adult relapsed and refractory (r/r) B-cell acute lymphoblastic leukemia (B-ALL), the CD19-targeting CAR-T product Tisagenlecleucel (Kymriah) represents a major milestone (10, 11). To date, six CAR-T cell products, are approved by the US Food and Drug Administration for the treatment of r/r hematologic malignancies. Despite these advances, disease relapse continues to be a major shortcoming of CAR-T cell therapy, with CAR-T cell persistence identified as one of the key contributing factors (12). Although there is ample evidence regarding the importance of CAR-T durability, the molecular factors that influence persistence are not as clear (13, 14). In this context, persistence refers to the maintenance of CAR-T efficacy and viability beyond the stage of antigen clearance (15). CAR-T cells that are found to persist are generally characterized as “memory” phenotypes with strong proliferative and self-renewal capabilities (16–18). Many studies have aimed to increase our understanding of the transcriptional and epigenetic landscape associated with memory CAR-T cells, yielding significant insights that have yet to be translated into improved clinical products (19). Here we review the current understanding of how Wnt/β-catenin signaling shapes T cell differentiation from hematopoiesis to maturity and further discuss how its modulation has been applied to CAR-T cells to enhance treatment durability.

## 2 T-cell development and memory formation

### 2.1 Thymic T cell development

**Figure 1:**
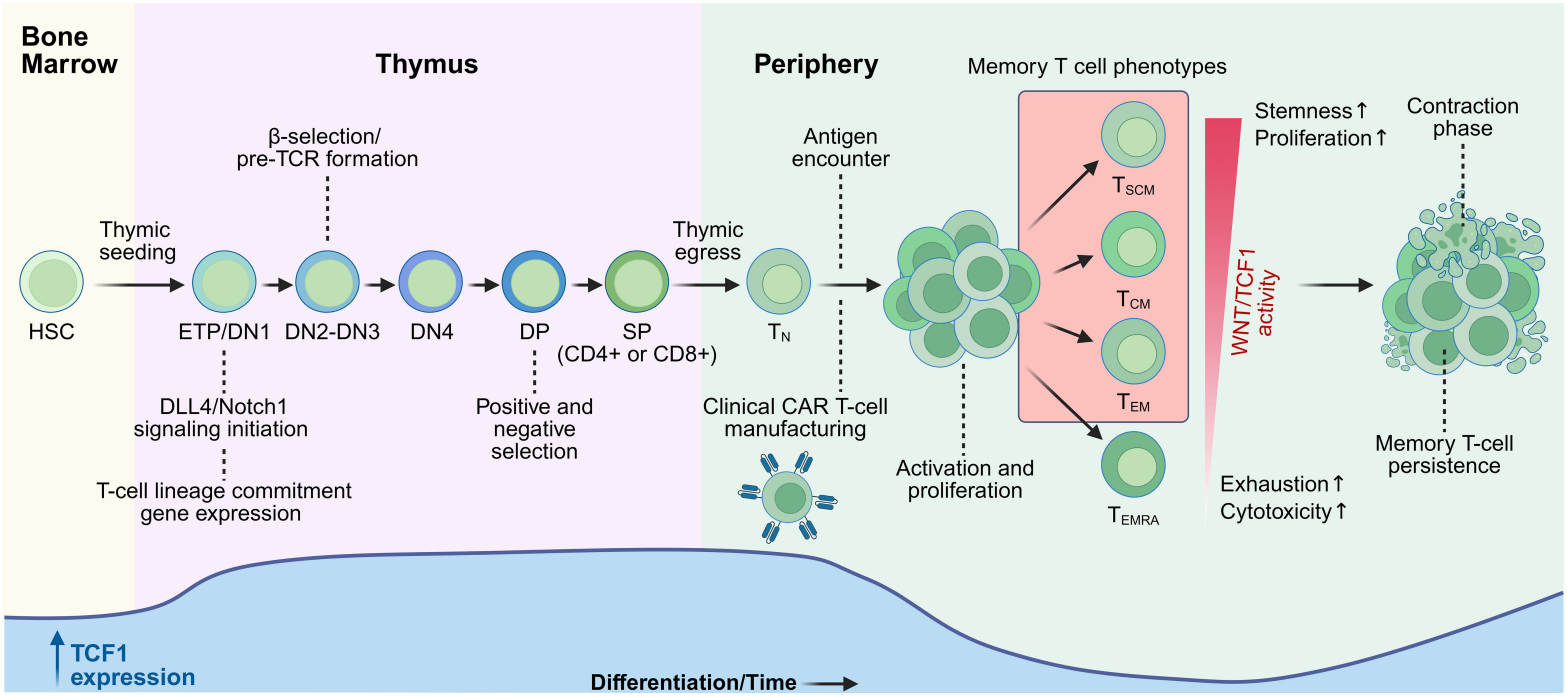
TCF1 expression varies over time across T cell development from HSC precursors to mature T cells in the periphery. Beginning as hematopoietic stem cells (HSCs), precursor cells travel via the bloodstream to seed the thymus, where they progress from early T cell progenitors (ETPs) through double negative (DN) stages (DN1-4), the double positive (DP) stage where positive and negative selection occur, and finally the single positive (SP) stage where they commit to either a CD4+ or CD8+ cell lineage. Upon thymic egress, mature naïve T cells survey the periphery where they encounter antigens, leading to T cell activation and proliferation producing a mix of phenotypes: stem cell memory T cells (T_SCM_), central memory T cells (T_CM_), effector memory T cells (T_EM_) and effector memory re-expressing CD45RA T cells (T_EMRA_). These phenotypes are displayed from top to bottom in order of differentiation which corresponds to overall Wnt/TCF1 activity (red gradient). This proliferation phase is followed by a contraction phase where shorter-lived cytotoxic cells are lost and only memory cells persist. T cell factor 1 (TCF1) expression is depicted as a blue curve across all stages of T cell development showing increased levels after DLL4/Notch1 signaling in the thymus and a reduction following antigen encounter and T cell activation in the periphery. Memory cell phenotypes (presented by the red box) that survive the contraction phase and remain present at the latest timepoint shown on the scale correspond with increased levels of TCF1 expression. Figure created using https://www.biorender.com/.

Beginning in the bone marrow, hematopoietic stem cells (HSCs) give rise to common lymphoid progenitors that travel via the bloodstream to the thymus, a process called thymic seeding, establishing the early T cell progenitor (ETP) population (**Figure 1**) (20). ETPs progress through a series of developmental stages, from double negative (DN, CD8-CD4-) to double positive (DP, CD8+CD4+) before committing to a single positive (SP) phenotype, CD4+CD8- or CD4-CD8+. The DN stage is further subdivided into DN1-DN4, characterized by Delta-like ligand 4 (DLL4) induced Notch1 signaling which drives early-stage T cell specific differentiation, followed by successive developmental checkpoints, including β-selection and the formation of a pre-T-cell receptor (pre-TCR) at DN3 (**Figure 1**) (21, 22). Pre-TCR signaling determines whether developing thymocytes ultimately commit to either αβ or γδ lineages (21, 23); γδ T cells diverge into a separate lineage not covered in the scope of this review. Conventional αβ T cells progress to the DP stage where interactions with surrounding stromal cells can influence phenotypic commitment to either CD4+ helper or CD8+ cytotoxic T cells through TCR signaling strength and downstream transcription factor activation (21, 24, 25). A process of positive selection then tests the αβTCR affinity for self-peptides presented by major histocompatibility complexes (MHCs) on cortical epithelial cells (23), while an additional negative selection checkpoint eliminates thymocytes with excessively strong self-reactivity to prevent autoimmunity (23, 25). Together, these checkpoints ensure that only T cells with an optimal binding capacity proceed to mature T cells in the periphery (21, 24).

### 2.2 Peripheral T cell memory acquisition

Upon exiting the thymus, naïve T cells survey the periphery. After antigen encounter, T cells undergo an expansion and differentiation phase that produces both effector and memory populations (**Figure 1**) (26), although the precise order of this differentiation is still under investigation and highly debated within the field (27, 28). Following antigen clearance, a large majority (90-95%) of antigen-specific T cells are lost through a dynamically regulated contraction phase, leaving a small fraction that has acquired long-lived memory characteristics including enhanced self-renewal and the ability to mount a robust response to antigen re-encounter (26, 27, 29). The surviving pool of memory T cells is understood to be phenotypically and functionally heterogeneous, and the field has classified this diversity into subsets reflecting progressive states of differentiation (30, 31).

The five widely recognized T cell subsets are: naïve (T_N_), memory stem cell (T_SCM_), central memory (T_CM_), effector memory (T_EM_), and terminally differentiated effector memory re-expressing CD45RA (T_EMRA_)(**Figure 1**), defined through the expression of unique pairings of surface molecules like CD45RA, CD45RO, CCR7, CD62L, CD27, and CD95. T_N_ represent populations of T cells that have not yet encountered antigen and maintain a high degree of lineage plasticity. T_SCM_ populations are phenotypically similar to T_N_, and along with T_CM_, are generally considered the less differentiated memory phenotypes delineated by longer lifespans, greater self-renewal potential, and the ability to rapidly respond to familiar antigens. T_EM_ and T_EMRA_ populations represent the more terminally differentiated effector populations which have greater immediate cytotoxic potential but relatively shorter lifespans, and are more prone to exhaustion (30–32). However, these categories represent broad groupings that do not always accurately predict T cell behavior. Therefore, researchers have moved to incorporate further genetic, epigenetic, and metabolomic analyses with the goal of gaining a more comprehensive definition of T cell memory (30).

Among the transcriptional programs critical for shaping T cell memory, the Wnt/β-catenin signaling pathway stands out (33, 34). While both CD4+ and CD8+ T cell lineages generate memory populations, the role of Wnt signaling in this context is more extensively studied in the CD8+ compartment. Although studies have implicated Wnt/β-catenin signaling in CD4+ T cell biology, the findings are less consistent relative to CD8+ cells and warrants further research (33, 35, 36).

## 3 The role of Wnt signaling in immature and mature T cells

### 3.1 Canonical Wnt/β-catenin signaling in hematopoiesis and thymic T cell development

Wnt signaling has been extensively studied in the context of T cell biology and development (34, 37, 38). The 19 lipid-modified glycoproteins that comprise the Wnt ligand family mediate signaling either through canonical or non-canonical routes (3, 4, 7). The non-canonical route, operating independently of β-catenin, regulates important aspects of T-cell biology (34, 38), but the canonical pathway is more extensively characterized with regard to T cell memory making it the primary focus of this review.

Canonical Wnt/β-catenin signaling involves Wnt1 and Wnt3a binding to membrane bound Frizzled receptors (FZD) and their co-receptor, low-density lipoprotein receptor-related protein 5/6 (LRP5/6), activating downstream signaling through β-catenin nuclear mobilization (4, 5). The transcription factors T cell factor 1 (TCF1 encoded by *TCF7*) and lymphoid enhancer binding factor 1 (LEF1 encoded by *LEF1*) are some of the most extensively defined downstream effectors of the canonical Wnt signaling cascade, playing key roles in T cell memory generation (33, 34). In the absence of Wnt signaling, TCF1 and LEF1 function as transcriptional repressors. However, upon Wnt activation and β-catenin stabilization, these proteins directly bind β-catenin and instead promote the activation of target genes (5, 39).

The effects of Wnt signaling in HSCs are nuanced and highly dependent on factors like signaling strength, context, and stage, with signaling input stemming from cell-intrinsic components as well as from the environmental niche (37, 40, 41). At low levels of activation, Wnt promotes multipotency, but excessively high levels can disrupt differentiation (37, 40, 41). Similar to their HSC precursors, T cell progenitors in the thymus have demonstrated a strong dependence on Wnt signaling for proper development (37), first shown through an *in vitro* study reporting impaired thymocyte differentiation with diminished Wnt/TCF1 activity (42). Multiple studies also assessed bone marrow progenitors deficient in TCF1, and found a reduced number of ETP populations in the thymus, identifying TCF1 as both a direct target downstream of Notch signaling and a prerequisite for Notch responsiveness in pre-thymic progenitors (43, 44). Consistent findings were reported in other studies utilizing *Tcf7*-/- mice, confirming TCF1 as critical for normal thymic T cell development, while LEF1 was found to provide a more compensatory and partially redundant role (45–47). Building on the importance of TCF1-driven gene transcription in lineage differentiation, other studies have shown that TCF1 regulates T cell lineage associated epigenetics (48), and is capable of binding and reversing H3K27me3 repressed chromatin, thereby acting as a direct regulator of stemness associated gene transcription during early T cell development (48). The documented similarities between memory T cell gene expression profiles and that of long-lived HSCs (49) suggests that the role of Wnt in regulating memory is not necessarily restricted to development, and may also apply to mature T cells (33, 50).

### 3.2 Canonical Wnt/β-catenin signaling promotes memory formation in mature T cells

Although Wnt signaling has been extensively studied in immature T cells, its role in mature T cells is less clear. Gattinoni et al. were the first to demonstrate that activation of the Wnt/β-catenin pathway plays an important role beyond thymic development, showing that glycogen synthase kinase-3 beta (GSK-3β) inhibition by TWS119 in mature CD8+ T cells blocked terminal effector differentiation and promoted the accumulation of less differentiated memory phenotypes like T_SCM_ and T_CM_ (32, 50). *TCF7* and *LEF1* expression is highest in less differentiated populations and declines progressively upon antigen encounter as the cell differentiates to more effector phenotypes, a pattern found in both human and murine models (**Figure 1**) (32, 51–53). This relationship has been more extensively characterized in CD8+ T cells, although broadly analogous trends are also found in CD4+ T cells but with much less consistency (32, 33, 36, 51, 52).

Within memory populations, TCF1 expression is found to be heterogeneous. Defined by Kratchmarov et al. into three populations, TCF1 high, TCF1 intermediate, and TCF1 low, the TCF1 high population exhibited quiescence and limited effector functions. Representing a large portion of the memory pool, TCF1 intermediate cells demonstrated rapid effector function, while TCF1 low cells were characterized by low self-renewal capacity and enriched among effector memory populations. Critically, reactivation of TCF1 high populations gave rise to both populations of TCF1 high and TCF1 low cells, directly demonstrating their superior self-renewal capacity relative to TCF1 low populations which were incapable of producing TCF1 high progeny (54). *Tcf7*-/- mice further confirm the importance of TCF1 for the maintenance of mature CD8+ T cells in the periphery. TCF1 deficiency led to diminished lifespans and impaired central memory formation in CD8+ cells, with mechanistic analysis revealing a critical link between TCF1, anti-apoptotic molecules like Bcl-2 and IL-2Rβ, and Eomesodermin (Eomes) (55). On an epigenetic level, memory T cells differ from exhausted T cells in chromatin accessibility at key loci, where super-enhancer activity proximal to memory associated genes like *TCF7* and *IL2RA* are enriched in memory populations compared with exhausted T cells (56). Together, these findings support Wnt/TCF1 signaling as critical for memory T cell generation in the periphery, with direct relevance to immune-based therapies like CAR-T cells.

## 4 Wnt signaling in CAR-T-cell therapy

### 4.1 CAR-T designs and downstream signaling activation

Because CAR-T cells are derived from a patient’s own T cells, the molecular programs governing memory in endogenous T cells may also apply to CAR-T cell biology and manufacturing. The chimeric antigen receptor (CAR) is a genetically engineered surface construct designed to bind a target antigen and activate endogenous cytotoxic T-cell functions (57). Structurally, the CAR construct can be broken down into a ligand binding domain, a spacer domain, and a transmembrane domain (57–59). The CAR is responsible for signaling initiation, but numerous factors can influence the strength of activation (57, 60–64). Successive CAR generations have incorporated costimulatory domains, commonly CD28 and 4-1BB, to improve overall efficacy through enhanced persistence or signaling strength. Studies have shown the variable consequences of utilizing certain costimulatory domains over others (65), leading to an array of novel CAR designs in preclinical stages, reviewed at length in other publications (66).

Despite numerous modifications to CAR designs across generations, the core signaling pathway remains consistent. In its simplest form, the activation domain of first-generation CARs is made up of a portion of the CD247, also known as T-cell receptor zeta (ζ) or CD3ζ. The zeta chain used in CAR-T cell constructs has three immune-tyrosine activation domains (ITAMs) in the cytoplasmic region, which are phosphorylated by lymphocyte-specific protein tyrosine kinase (Lck) upon antigen recognition (57, 62). This phosphorylation initiates recruitment of zeta-chain-associated protein kinase 70 (Zap-70), which in turn activates downstream signaling cascades including those mediated by IL-2-inducible tyrosine kinase (Itk) (67). ITAM mediated activation in combination with costimulatory signals from CD28 and 4-1BB in second generation CARs produce unique signaling behaviors compared with native TCR activation (62). Antigen recognition by a CAR-T cell initiates the secretion of perforin and granzyme B which initiate apoptosis of nearby target cells (68), and facilitates the activation of the multiple downstream signaling pathways (62).

Broadly, CAR engagement is found to activate the PI3K/AKT pathway, the nuclear factor kappa-B (NF-κB) pathway, the nuclear factor of activated T cells (NFAT) pathway, and the activator protein-1 pathway (AP1), collectively influencing T cell behavior (62, 65). Activation also promotes the release of pro-inflammatory cytokines like IL-2, TNF, and IFNγ responsible for enhancing cytotoxic immune functions (62). Relative to what is known about TCR activation, there remains a lack of clarity on the unique combination of signaling events that occur with CAR engagement, likely differing with each new design modification.

### 4.2 Wnt/TCF1 as a correlate of CAR-T clinical response

Consistent engagement of the CAR domain leads to progressive differentiation that can ultimately result in exhaustion, a core obstacle in CAR-T cell therapy (69, 70). Early clinical evidence from a cohort of patients with chronic lymphocytic leukemia (CLL) who received CD19 directed CAR-T therapy revealed that patients who achieved complete response (CR) or sustained remission exhibited enrichment of memory-associated gene expression at the time of infusion compared with non-responders. Conversely, non-responders showed infusion products with higher levels of exhaustion-associated gene expression (15). A long-term follow up study in a similar CLL cohort showed that CAR-T cells remained detectable in patients with CR, and strikingly, the dominant phenotype among circulating CAR-T cells at late timepoints was a cytotoxic CD4+ subset (71). Consistent results were also found in a study of patients with large B cell lymphomas receiving axicabtagene ciloleucel, a commercial CD19-directed CAR-T product, where those achieving CR harbored notably higher levels of memory related gene expression in CD8+ T cells when compared with cases of partial response or progressive disease (72). Interestingly, a large-scale analysis of pre-manufacture T cells from patients with B-cell malignancies revealed that the *TCF7* regulon was a key determinant of CAR-T response, found in both naïve and effector T cells among patients with favorable clinical outcomes (73). More recently, a study correlating patient outcomes with post-infusion CAR-T phenotypes in a non-Hodgkins lymphoma (NHL) cohort treated with commercial CAR-T products identified populations enriched in complete responders, including CD8+ CAR-T cells positive for programmed cell death protein 1+ (PD-1+) at 14 days post-infusion (74). This subtype was further analyzed and subdivided identifying stem-like populations positive for TCF1 (CD8+PD-1+TCF1+) and effector subsets that were specifically associated with favorable clinical outcomes (response and progression-free survival) (74).

Despite the clinical evidence correlating TCF1 expression, memory, and persistent CAR-T cells, overexpressing TCF1 in CAR-T cells revealed that TCF1 regulation alone was insufficient to retain memory programming or enhance CAR-T potency (75). Instead, the transcription factor FOXO1 was revealed as an integral driver of CAR-T cell memory and exhaustion resistance over TCF1 (75, 76). Additionally, a study of CD8+ CD19-directed CAR-T cell responses in pediatric and young adult patients with acute lymphoblastic leukemia revealed, through longitudinal analyses of DNA methylation in post-infusion CAR-T cells, extensive epigenetic remodeling that included specific suppression of *TCF7* and *LEF1* as part of the progressive acquisition of an exhaustion-related methylation profile (77). These findings highlight the Wnt/TCF1 axis as a strong clinical correlate of CAR-T responses, but not necessarily its primary driving factor.

### 4.3 Targeting the Wnt/TCF1 axis during CAR-T cell manufacturing

**Figure 2:**
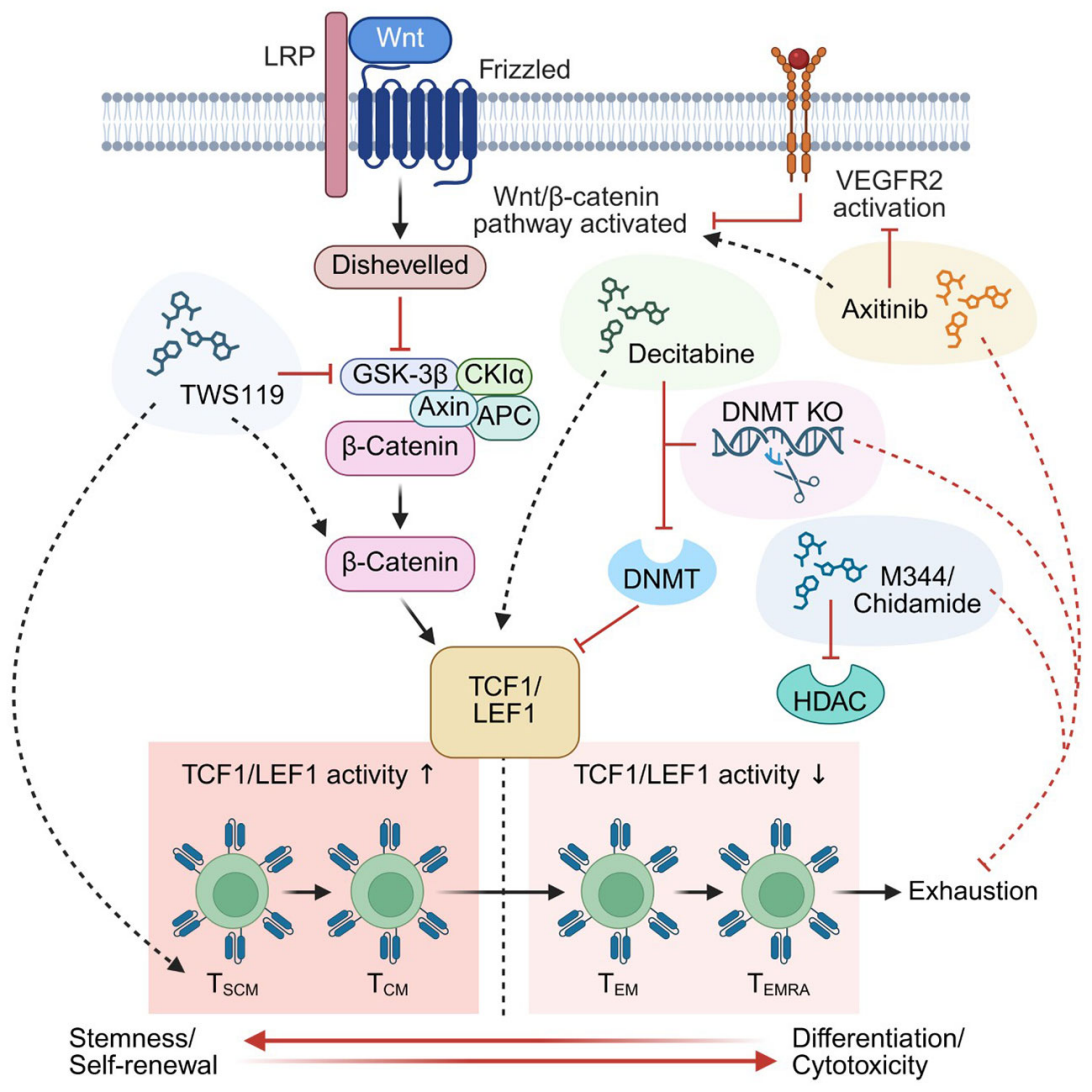
Wnt/TCF1 directed CAR-T cell manufacturing strategies. Wnt/β-catenin signaling is shown in activated form where the Wnt ligand is bound and the destruction complex is inactivated allowing β-catenin to progress and interact with downstream binding partners TCF1/LEF1. Below TCF1/LEF1 are two broad outcomes of their activation, where TCF1/LEF1 high activity has been shown to correlate with less differentiation CAR-T cell phenotypes (T_SCM_ and T_CM_) while TCF1/LEF1 low activity is more associated with effector populations that represent increased differentiation states (T_EM_ and T_EMRA_) more prone to exhaustion. VEGFR2 receptor activation is shown to the right of Wnt receptors LRP and Frizzled. Solid black lines represent direct interactions or binding partners, and solid red lines represent direct inhibitory actions. Dashed lines show indirect outcomes where black lines indicate activation, and red lines show inhibition. Drug based intervention methods are shown by a generic chemical symbol that do not represent the real chemical structure (TWS119, Decitabine, Axitinib, and M344/Chidamide) and CRISPR/Cas9 methods are shown by the scissor symbol (DNMT CRISPR/Cas9 KO). Figure created using https://www.biorender.com/.

Clinical evidence linking Wnt/TCF1 signaling activity to CAR-T persistence has motivated growing efforts to target this pathway during manufacturing to enhance CAR-T memory formation and persistence. Sabatino et al. demonstrated that pharmacological activation of Wnt/β-catenin signaling via the GSK-3β inhibitor TWS119, applied in conjunction with cytokines IL-7 and IL-21, promoted T_SCM_ phenotypes in clinical-grade CD19-directed CAR-T cells with superior characteristics compared with conventionally manufactured products (**Figure 2**) (78). These findings were consistent with pre-clinical data showing GSK-3β inhibition enriches for T_SCM_ populations during activation, but cannot reverse differentiation in committed effector cell populations, supporting the early use of this inhibitor at the CAR-T manufacturing stage (79).

Building on evidence that T cell exhaustion is, in part, driven by *de novo* DNA methylation (80), a study by Prinzing et al. demonstrated that CRISPR-mediated deletion of DNA methyltransferase (DNMT) 3 alpha conferred exhaustion resistance in CAR-T cells by hindering the repression of genes regulating multipotent cell potential (**Figure 2**) (81). Genome-wide methylation profiling provided a defining catalogue of exhaustion-related genes, revealing that *TCF7* and *LEF1* were among the first stemness-associated loci to be silenced in the progression towards effector cell phenotypes and exhaustion (81). These results support that early suppression of this signaling axis may play a causal role in driving CAR-T cell dysfunction. Wang et al. took a more clinically translatable approach by applying low-dose decitabine, an FDA-approved DNMT inhibitor, during ex vivo manufacturing to achieve similar preservation of memory-associated gene activity (*TCF7* and *LEF1* among others) and enhanced anti-tumor activity *in vivo* (**Figure 2**) (82).

Additional pharmacological evidence for Wnt-regulated memory programming in CAR-T cells comes from high-throughput screening of chromatin-modifying agents. The use of histone deacetylase inhibitors (HDACi) M344 and chidamide, a sub-type selective HDACi specifically inhibiting HDAC 1, 2, 3, and 10, were found to promote memory in CAR-T cells and confer exhaustion resistance (**Figure 2**) *in vitro* and *in vivo*, upregulating *TCF4*, *LEF1* and *CTNNB1* which activate the canonical Wnt/β-catenin pathway leading to increased expression of downstream targets like *TCF7* (83). Separately, pharmacological inhibition of vascular endothelial growth factor receptor 2 (VEGFR2) using Axitinib during manufacturing increased Wnt/β-catenin activity in CAR-T cells leading to reduced exhaustion and enhanced antitumor efficacy in a B-ALL model (**Figure 2**) (84). Collectively, these findings point to the growing feasibility of targeting the Wnt/β-catenin pathway during ex vivo manufacturing as a clinically applicable strategy for improving CAR-T cell persistence and durability (85).

## 5 Discussion

Recapitulating T-cell stem-like memory states prior to CAR-T infusion offers a promising approach to enhance long-term durability. In this review, we highlight how Wnt/β-catenin signaling regulated memory formation and self-renewal, underscoring its potential to promote sustained antitumor responses. While TCF1 emerged as a consistent marker for memory-associated differentiation and for clinical outcomes, it operates within a broader regulatory network. Importantly, TCF1 overexpression alone was not sufficient to preserve memory, whereas other factors like FOXO1 were defined as more proximal drivers of CAR-T memory programming (75). The findings discussed here emphasize the need to move beyond TCF1 abundance alone, and to examine adjacent Wnt regulators that may play a role in persistence. Furthermore, the identification of crucial cytotoxic CD4+ CAR-T cell populations in patients with decade-long remissions (71) emphasizes the clinical relevance of CD4+ CAR-T cell persistence and highlights a gap in our understanding of how Wnt/TCF1 signaling regulates cell fate programs in this subset, which has received considerably less attention than CD8+ counterparts.

An important complexity not covered in this review arises from the role of Wnt signaling in the tumor microenvironment and across the tumor-immune interface. Whereas Wnt activation in T cells supports memory programming, tumor-intrinsic Wnt signaling promotes immune suppression associated with impaired CAR-T cell efficacy and infiltration (86). This distinction is important to consider when implementing therapeutic strategies to improve persistence and may reflect the predominant use of Wnt-directed therapeutic strategies implemented during CAR-T manufacturing rather than after infusion.

Significant gaps remain in our understanding of CAR-T memory formation. A deeper mechanistic understanding of how Wnt signaling integrates with these regulatory processes may help inform novel therapeutic strategies to improve CAR-T cell persistence and thus improve patient outcomes.
